# Measuring social integration, treatment, and mortality after substance use treatment: methodological elaborations in a 20-year follow-up study

**DOI:** 10.1186/s13104-025-07108-3

**Published:** 2025-01-21

**Authors:** Tove Sohlberg, Jessica Storbjörk, Peter Wennberg

**Affiliations:** 1 Department of Public Health Sciences, Stockholm University, Stockholm, Sweden; 2 Centre for Social Research on Alcohol and Drugs (SoRAD), Stockholm University, Stockholm, Sweden; 3 Department of psychology, Inland Norway University of Applied Sciences, Lillehammer, Norway; 4 Department of Global Public Health, Karolinska Institute, Stockholm, Sweden

**Keywords:** Long-term outcomes, Substance use treatment, Alcohol, Drugs, Sweden

## Abstract

**Objective:**

Alcohol and Other Drug (AOD) disorders cause substantial harm. Effective Substance Use Treatment (SUT) exists, but long-term outcomes remain inconclusive. This study used a 20-year prospective follow-up of 1248 service users entering SUT in Stockholm, Sweden, in 2000–2002 to elaborate on how different dimensions of long-term outcomes may be measured by register-based indicators. Baseline characteristics and attrition bias were explicated, and register-based outcomes were examined.

**Results:**

Register-based indicators are valuable, but they also have inherent limitations such as the lack of substance use data and inability to differentiate between un/met treatment needs and access. Significant variations in long-term outcomes were evident depending on which register-based indicator was used, and whether used in isolation or combinations. Six out of 10 service users were still alive after 20 years, but as many as 8 out of 10 of the survivors remained in treatment, and only two out of 10 had a stable economic situation. Hence, the register indicators identified only a few survivors, with stable economic and social situations, and without recent treatment contacts 20 years after treatment entry. The long-term outcomes were concerning and even more so when combining outcome dimensions.

**Supplementary Information:**

The online version contains supplementary material available at 10.1186/s13104-025-07108-3.

## Introduction

Alcohol and other drug (AOD) disorders cause substantial harm [1]. Substance use treatment (SUT) has favorable effects [2], but long-term outcomes remain inconclusive [2–4]. This is due in part to variations in data and outcome measures used across studies, and differences in populations studied [5, 6].

While some long-term follow-up studies examine abstinence or substance use, 10 to 50 years after treatment [7–12], many rely on mortality [13, 14]. Few include indicators of social integration [15, 16], although aspects such as social stability, networks, and employment are associated with long-term success [7, 11, 17, 18]. Other important measures linked to outcomes include co-occurring mental ill-health in people with substance use disorders (SUDs) [8, 19–22], and treatment utilization. Often remission takes time [23] and recurrent or long-term treatment can be needed [24–26]. Furthermore, SUD diagnoses in healthcare registries are oft-used indicators of AOD problems.

This article elaborates on how register data can measure not only mortality, but treatment utilization and social integration 20 years after entering SUT, and outcomes depending on which measure is used. Different outcome dimensions, as single measures and in combinations, were examined in a sample representative of a whole treatment system, as well as by substance groups (that differ in age). This adds to our knowledge, as many follow-ups, especially RCTs, apply a range of eligibility criteria and tend to exclude, for example, people in homelessness or with co-occurring mental illness [27, 28]; and long-term follow-up studies often focus on a single substance, e.g., opioids *or* alcohol [9–12], specific genders [12, 29], or particular treatment modalities [29, 30]. Major advantages of our project “*Recovered*,* in treatment or dead: a 20-year follow-up of women and men in Swedish substance use treatment”* were that it included a heterogeneous sample of male and female AOD users, including those using non-prescribed pharmaceuticals, entering the full range of SUT services in Region Stockholm (2.4 million inhabitants in 2020 [31]), and that it used a prospective design.

### Baseline in 2000–2002: Year 0 (Y_0_)

At baseline, 1865 adults were interviewed at SUT entry. The project [32](reference 32 includes a link to an English translation) aimed to represent the whole system by covering publicly funded medical and social services-based SUT: hospital-based inpatient care/detoxification, long-term residential and compulsory care, opioid agonist treatment (OAT), outpatient programs, and housing interventions. Information on SUT in Sweden is available elsewhere [33].

The structured one-hour interview [34, 35] used questions from the CIDI [36] to assess ICD-10 (3 + criteria) dependence (alcohol and/or main drug of choice) [37].

One-year and five-year follow-up interviews are described elsewhere [17, 28, 38, 39].

### Register-based follow-up in 2020: Year 20 (Y_20_)

Y_20_ included eligible patients/clients who had consented to register-based follow-up, and provided correct ID: s (*n* = 1248; flowchart in **Additional file S1**).

Y_20_ used register data retrieved from the National Board of Health and Welfare (NBHW) and Statistics Sweden (SCB). Depending on availability at the time of extraction, data were obtained for 2000–2019 or 2000–2020. In order to reflect long-term and stable outcomes, the measures examined refer to the last *five* years (2015–2020).

Information on diagnoses and healthcare-related SUT in 2000–2020 was extracted from the *National Patient Register* [40]. Pharmacotherapies were available from the *National Prescribed Drug Register* [41] (established in 2005). Compulsory care was retrieved from the *National Register of Care for Substance Abuse* [42], and mortality from the *Cause of Death Register* [43, 44] (2000–2020). Information on marital status, family life and income was obtained from *Statistics Sweden* for 2000–2019 [45].

Full details of the baseline questionnaire and registry operationalizations are provided in **Additional files S2–S3**.

### Statistical analyses

Uni- and bivariate analyses were performed to describe sample characteristics and attrition bias, and to elaborate on the outcome indicators. Chi-square tests (χ2) were used to assess significant differences across groups.

### Baseline characteristics and attrition

**Additional file S3** shows detailed baseline sample profiles. The follow-up sample (Y_20_) had a median age of 44 years at baseline. The majority were male. Few had a stable social and economic situation.

Alcohol dependence was the most commonly reported sole dependence (54.6% of those followed-up). In addition (not shown), opioid dependence was most prevalent among those dependent on another drug only and/or in combination with alcohol dependence (45.1%), followed by dependence on amphetamines (21.5%), sedatives/psychotropics (13.0%), cannabis (13.7%), and “other.” The median age differed across these groups: alcohol dependence only (51 years), illicit/prescription drug only (35 years), alcohol combined with another drug (38 years), and 45 years among those that did not meet diagnostic criteria for dependence at Y_0_.

About half (≈ 53%) of the Y_20_ sample was recruited from SUT within the healthcare sector, the other half from SUT provided by social services. Most had SUT experiences *prior to* baseline. Few had been in OAT, which was only available as Methadone maintenance and strictly regulated in the early 2000s [46].

The Y_20_ sample did not differ significantly from the baseline sample (Y_0_), which suggested good representativity for this long-term follow-up (**Additional file S3**).

### Outcome dimensions

#### Mortality

The broad usage of mortality data may be understood by its reliable, definitive and objective character.

We note that 42% (*n* = 526) had deceased by the end of 2020: 58.6% of those initially dependent on alcohol only died during the follow-up, followed by drug dependence only (18.1%), no dependence (15.4%), and combined dependence (8%) (*p* < 0.001).

Changing focus to premature death (Table 1), defined in Sweden as death before the age of 65 [47], we see that the majority (63%) of the deceased did not reach Swedish 65-year retirement age. The highest rates of premature death were among individuals with sole or combined drug dependence. A majority of the deceased that did not meet 3 + dependence criteria reached retirement age.

**Table 1:** Normal versus premature age at death (%) between 2000 and 2020, by type of substance dependence at baseline.^a^

Dead after 20 years (Y_20_)	Normal age at death (≥ 65) *n* = 193	Premature death (≤ 64) *n* = 333	Median age at death
Patients/clients… …with alcohol dependence, only (*n* = 308)	42.2	57.8	63
…with illicit/prescription drug dependence, only (*n* = 95)	9.9	90.5	44
…with combined dependence on both alcohol and another drug (*n* = 42)	14.3	85.7	51
…that did not meet diagnostic criteria for alcohol or drug dependence (*n* = 81)	59.3	40.7	69
Total (*n* = 526)	36.6	63.4	57

^a^ Dependence diagnoses based on ICD-10 criteria (3+), as estimated by the CIDI structured interview at baseline

**Table 2:** Service users’ substance use treatment (SUT) at follow-up (Y_20_; 2016–2020), in total and by substance dependence ^a^ at baseline 2000–2002, register data, column percentages.^b^

Situation at 20-year follow-up (Y_20_)	Only alcohol dependence *n* = 308	Only drug dependence *n* = 95	Alcohol and drug dependence *n* = 42	No AOD dependence ^c^ *n* = 81	Sig. level Chi^2^-test	Total alive in 2016–2020 *n* = 721
**No SUT for SUD**						
No in- or outpatient alcohol or drug treatment	15.2	13.9	21.7	38.3	0.001	18.4 ^d^
**Treatment**,** in total**	73.6	81.7	72.6	39.6	0.001	73.0
Outpatient for alcohol/drug diagnosis…without AOD-related medication	79.8	70.2	70.7	51.9	0.001	71.9
…with alcohol-related medication	55.2	17.0	36.4	17.6	0.001	34.1
…with drug-related medication– OAT	0.9	39.7	20.0	2.9	0.001	18.5
Inpatient care for alcohol/drug diagnosis, and/or compulsory care	50.8	42.9	56.6	34.2	0.006	47.0
Co-morbidity: Inpatient with AOD diagnosis and psychiatric diagnosis	28.8	27.7	22.1	25.0	ns	27.0

^a^ Dependence diagnoses based on ICD-10 criteria (3+), as estimated by the CIDI structured interview at baseline

^b^ Column percentage do not add to 100% due to overlapping treatment episodes

^c^ No AOD dependence refers to individuals who did not meet diagnostic criteria for alcohol or drug dependence (see footnote a)

^d^ No SUT and SUT in total do not add to 100% due to differences in the concepts (see **Additional file S2**)

**Table 3:** Social integration at follow-up (2015–2019) by different criteria, column percentages (%), in total and by substance use treatment (SUT) groups

	SUT in 2015–2019					
	No SUT (*n* = 88)	Only outpatient care or AOD medication (*n* = 211)	Only inpatient or compulsory care (*n* = 204)	Both outpatient & inpatient care (*n* = 185)	*p*-value	Total (*n* = 688)
**Income & labor market ties**					0.001	
Economically excluded	33.3	37.7	57.9	67.3		50.9
Weak economic situation	36.7	44.7	26.3	15.0		30.4
Stable economic situation	30.0	17.5	15.8	17.8		18.7
**Income and family/social ties**					0.001	
Economically excluded + living with another adult	15.3	17.1	14.4	25.1		19.1
Economically excluded + not living with another adult	11.9	27.3	53.6	36.9		33.5
Weak economic situation + living with another adult	20.3	21.5	8.2	11.2		15.6
Weak economic situation + not living with another adult	23.7	14.1	6.2	8.4		11.9
Stable economic situation + living with another adult	25.4	14.6	7.2	12.8		13.9
Stable economic situation + not living with another adult	3.4	5.4	10.3	5.6		6.1
**NEET**,** adapted**	34.1	4.2	42.2	2.7	0.001	18.9
**Social assistance**	49.3	50.0	48.6	69.7	0.001	55.8
**At-risk-of-poverty**,** relative**						
< 60%	29.6	6.6	18.9	16.6	0.001	14.7
< 50%	7.5	4.6	8.9	10.6	ns	7.8

Premature death may also refer to death before the average age of death in a given population [22]. In 2019 (before covid-19) life expectancy in Sweden was 83.1 years (84.7 for women, 81.4 for men) [48]: A majority of the deceased died prematurely, and the median age at death was much lower than expected.

### Continued treatment utilization and AOD diagnoses

About one-fifth (18.4%) of the survivors (*n* = 722) had no records of healthcare-based SUT for SUD 2016–2020 (Y_20_), leaving a vast majority with AOD diagnoses and continued need of interventions 15–20 years after entry (Table 2). SUT at Y_20_ was most common among those initially drug dependent.

Outpatient treatment without AOD-medication was most often used (71.9%), followed by inpatient and/or compulsory care. About one-third had received alcohol-related medication. Almost 40% of those with a history of drug dependence received OAT at Y_20_. Most of those dependent on alcohol at Y_0_ had used outpatient care with pharmacotherapy at Y_20_. Alcohol-related pharmacotherapy was common also in the other groups. Those who did not meet diagnostic criteria for dependence at baseline were regular users (over 70%) of outpatient treatment at follow-up. 27% received inpatient care for psychiatric co-morbidity.

### Social integration

Integration in society is difficult to assess by register data, due to missing information on e.g., social network and subjective satisfaction.

Starting with a definition and operationalization guided by the NBHW [49] and Alm [16] we elaborated on income in combination with labor market ties (Table 3). This indicator yielded a high proportion considered *economically excluded* at 20 years (50.9%). Less than one-fifth had a *stable economic situation*. Combining this social integration measure with continued treatment need, reveals that individuals without SUT at Y_20_ more frequently (30.0%) had a *stable economic situation*.

Next, we combined income and labor market ties with family/social ties: *living* or *not* living *with another adult*. Very few had a stable economic *and* social situation (13.9%). Most were categorized as economically and socially excluded (33.5%) when using this measure.

When elaborating on NEET (Not in Education, Employment or Training) [50], the exploration showed that such social exclusion was more common among those who at Y_20_ had either received inpatient and/or compulsory care, or no SUT for SUD at all.

Finally, a measure of relative poverty or low economic standard was examined: *Persons at-risk-of-poverty or social exclusion* was defined as a total income below 60 or 50% of the country’s median income, as well as the percentage receiving economic social assistance. The at-risk-of-poverty rate (14.7%) aligned with overall population Figs. (14,6%; mean for 2015–2019 [51]). However, 55.8% had received means-tested social allowances via social services at least once in the last five years. Combined with continued care, those without SUT at Y_20_ appeared to have the worst financial situation.

In conclusion, the different indicators provide different estimates of social integration at Y_20_, and significant associations with continued treatment use.

## Discussion

This study demonstrated significant variations in long-term outcomes depending on which register-based indicator was used, and whether used in isolation or combinations.

Elaborating on oft-used mortality measures indicated death rates (42%), and elevated rates of premature death, especially among individuals with a history of drug dependence. This aligns with the increase in drug-induced mortality in Sweden [46, 52]. However, the elaboration on treatment indicators suggests that mortality may have been mitigated by the widespread use of OAT by 2020 [14, 53].

In elaborating on who still has AOD diagnoses– often used for identifying SUD in registry-based research– we note that the majority (81%) of the survivors received SUT also 15–20 years after treatment entry. This is likely to be an underestimate since register-based information on SUT via social services is unavailable. The high occurrence of social assistance also suggests that at least half of the survivors were in contact with social services at follow-up.

While these outcome indicators appear high, they are nonetheless within the realm of plausibility: Remission takes time [23], multiple treatment episodes are often necessary [24–26], and SUDs are increasingly understood to require long-term or continuing treatment in order to prevent relapse and extend abuse-free periods [4, 54]. The reinforcement of disease/brain disease models also in Sweden during the follow-up [55], supports increased promotion of pharmacotherapy [56, 57]. That may explain why more than half of those with a history of alcohol dependence remained on alcohol-related medications at Y_20_. Treatment for psychiatric comorbidity was also observed for about one-third [12, 58]. This topic is highly debated in Sweden and is driving reforms to transfer all treatment responsibility to healthcare [59].

Hence, register-based service utilization data provides valuable outcome information, but has inherent limitations. Apart from the lack of a social services register in Sweden, the healthcare registers available do not provide data on substance use levels and patterns, and cannot differentiate between un/met needs or SUT access. As a result, continued treatment use may be perceived as either a failure or a favorable outcome. While extended support is beneficial, ultimately individuals should be able to lead a problem-free life without formal support.

The situation is further complicated when attending to indicators of social integration, and individuals may transition between social inclusion and exclusion [15]. These indicators suggest poor outcomes as half of the survivors were economically excluded after 20 years; close to 70% among those who also had used in- and outpatient SUT. Conversely, register data could identify smaller groups of survivors (25–30%) out of treatment and with a stable economic and/or social situation.

This is important since the overarching treatment goal ideally is not merely to eliminate disorders but to enable improved functioning. This elaboration found that combining different outcome dimensions significantly reduced success rates. Six out of 10 service users survived, but as many as eight out of 10 of the survivors remained in treatment. Only two out of 10 had a stable economic situation. Factors positively associated with abstinence, e.g., a stable social situation [11, 17] and labor market connections [11, 15, 17, 60], were a reality for only a few. This representative treatment system sample was rather vulnerable from the outset. Nevertheless, elaborating on and combining long-term outcome indicators suggest concerning treatment system performance.

### Limitations

Limitations include the lack of register-based information on: substance use; social networks beyond marital status/family type; and social services-based SUT. The proportion still in SUT is thereby probably underestimated, and uncertainties remain regarding interpretations and implications of social circumstances.

## Electronic supplementary material

Below is the link to the electronic supplementary material.

Supplementary Material 1

## Data Availability

The dataset analysed during the current study are not publicly available due to privacy or ethical restrictions but are available from the corresponding author on reasonable request.

## Abbreviations

AOD: Alcohol and other drugs
CIDI: The Composite International Diagnostic Interview
ICD: The International Classification of Diseases
NBHW: The Swedish National Board of Health and Welfare (NBHW)
NEET: Not in education, employment or training
OAT: Opioid agonist treatment
SCB: Statistics Sweden
SUD: Substance use disorder
SUT: Substance use treatment
Y_0_: Year 0, baseline
Y_20_: Year 20, at follow-up
