# Supplementary material for: Measuring social integration, treatment, and mortality after substance use treatment: methodological elaborations in a 20-year follow-up study
Source: BMC Res Notes. 2025 Jan 21;18:27. doi: 10.1186/s13104-025-07108-3 (PMC11749105; doi:10.1186/s13104-025-07108-3)
Supplement: Supplementary file 1 — Supplementary Material 1 [file 13104_2025_7108_MOESM1_ESM.docx]

# Electronic supplementary material ^[[1]](#footnote-1)^

## S1: Additional file 1: Flowchart

Y_0_

Baseline

N=1865

Excluded (n=58)

- Incorrect ID (n=28)
- Other reasons (n=30)

Final sample

(n=1248)

Y_20_

20-year follow-up

Consented to follow-up via registers

(n=1306)

## S2: Additional file 2: Register-based measures

Register data were obtained from the National Board of Health and Welfare (NBHW) and Statistics Sweden (SCB). The following measures were generated for the 20-year follow-up (Y_20_).

| **VARIABLE** | **SOURCE AND OPERATIONALIZATION** |
| --- | --- |
|  | ***Information derived from the National Patient Register (40); covering the years 2000–2020; provided by the NBHW and using ICD-10 (37).*** |
| *SUT for SUD* | *SUT* (Substance use treatment utilization) refers to *inpatient* and *outpatient* treatment for a substance use disorder (SUD). A main diagnosis is coded by the attending physician. Alcohol use disorders (AUD) are measured by the ICD-10 code F10, and drug use disorders referred to: F11 (opioids), F12 (cannabis), F13 (sedatives, hypnotics or anxiolytics), F14 (cocaine), F15 (other stimulants, including amphetamines) and F16 (hallucinogens). The different drug diagnoses were combined into a single variable measuring treatment for SUD. Thus, SUT here is treatment for an alcohol and/or drug diagnosis in a health care-based inpatient or outpatient setting. |
| *Treatment for dependence* | *Treatment* was measured as having received health care, including psychiatric treatment, AOD-related medication or compulsory care due to diagnosed alcohol and/or drug dependence. In the register, alcohol dependence was measured by the ICD-10 code F10.2 (alcohol dependence syndrome). Drug dependence referred to patients who had been diagnosed with one or more of the following ICD-10 diagnoses: F11.2 (opioid dependence), F12.2 (cannabis dependence), F13.2 (dependence on sedatives, hypnotics or anxiolytics), F14.2 (cocaine dependence), F15.2 (dependence on other stimulants, including amphetamines) and F16.2 (dependence on hallucinogens). |
| *OAT* | Opioid agonist treatment (OAT) with buprenorphine or methadone, was measured separately from *Treatment* above. |
| *Psychiatric comorbidity* | The diagnoses included were based on a combination of severity from a societal or individual perspective and included the full diagnostic groups: diagnoses of schizophrenia (ICD-10 code F20X) and other psychoses (F23X), bipolar affective disorder (F31X), and depression (F32X) in the National Patient Register, were combined into the variable *psychiatric diagnosis.* Thus, we did not include anxiety, strongly correlated with SUD, nor mental conditions like neuropsychiatric disorders and other personality disorders. We have also not included substance use disorders in this category for obvious reasons. |
|  | ***The National Prescribed Drug Register (41); established in July 2005; from NBHW*** |
| *AOD-related medication* | Pharmacotherapy included medications prescribed for alcohol-related problems (disulfiram, acamprosate, naltrexone, nalmefene) and opioid dependence (methadone, buprenorphine). These medications were combined into *AOD-related medication* in 2005–2020. |
|  | ***National Register of Care for Substance Abuse (42); from NBHW*** |
| *Compulsory care* | Measures from the register of the Care of Substance Abusers (Special Provisions) Act (LVM) register (42) (2000–2020) referred to those who were committed to *compulsory care,* including immediate detention, for AOD-problems at least once during the period 2016–2020. The numbers in this category were low and compulsory care was therefore combined with and/or *inpatient care* for alcohol and/or drug problems (*Treatment*) in the current article. |
|  | ***Cause of Death Register (43,44); from NBHW*** |
| *Mortality* | *Deceased respondents* were found in the Cause of Death Register (covering the years 2000–2020). |
|  | ***LISA, Longitudinal integrated database for health insurance and labour market studies (45); from Statistics Sweden (SCB)*** |
| *SES (and marital status)* | *Socioeconomic status (SES)* was based on information from Statistics Sweden (2000–2019) on declared income, and family type (categorized in the registers as DekLon, FamTyp). Declared income included salary or pension declared to the Swedish tax authorities. Family type included different combinations of being married/cohabiting with or without children or living alone (without another adult) with or without children. This variable was dichotomized as living with another adult or not. |
| *Income & labor market ties* | Declared income (see SES above) in combination with labor market ties was defined and operationalized with guidance from the NBHW (49) and Alm (16). An average (mean) price base amount (consumer price index) for 2015–2019 was calculated (3 800 EUR) and used to categorize participants into those who at Y_20_ had a *stable economic situation,^[[2]](#footnote-2)^* corresponding to a declared income of at least 3.5 price base amounts or studying; a *weak economic situation,* corresponding to a declared income between 0.5–3.5 or economical support for job search at the Swedish Public Employment Service. Those with a declared income below 0.5, early retirement, or social allowances/assistance were considered as *economically* *excluded.* |
| *NEET* | The measure “not in education, employment or training” (NEET) (50) was based on information from Statistic Sweden (2000–2019) on total income. This variable included not only salary and pension, but also sickness benefits, social assistance, parental and study allowances, and unemployment benefits (categorized in the registers as Raks_ArbLosInk, Raks_ForTidInk, Raks_AldPensInk, Raks_StudInk, Raks_UtbBidrInk, Raks_VardInk, Raks_SjukInk, Raks_EkBisInk, Raks_ForvInk). These variables were combined into one variable measuring income (Raks_SummaInk). NEET is mainly used for young adults, but is also relevant for subgroups with weak ties to the labor market. |
| *At-risk-of-poverty* | The two measures of *at-risk-of-poverty* were calculated as having a relative income below 50% or 60% of the national median income (51) . Firstly, the median income for the years 2015–2019 was collapsed, and secondly, an average (mean value) was calculated (260 700 SEK). Individual income was measured as the total sum of different sources of income (see NEET above). Typically, such measures of relative poverty are calculated after redistribution through taxes and social transfers. The extracted register data did not allow the calculation of income at household level or after tax deduction. Instead, crude measures of those with individual-level incomes below both 60 and 50 percent of the median income were used. |
| *Social assistance* | *Social assistance* (categorized in the register as socbidr_tot) formed a separate group and included having received means-tested financial benefits from the social services at least once during 2015–2019. |

## S3: Additional file 3: Sample characteristics at baseline, interview measures, and attrition bias

**Table:** Sample characteristics ^a^ at baseline (Y_0_) in 2000–2002, for the total sample, and among those lost to follow-up and those followed-up in year 2020 (Y_20_)

|  | **Y_0_** |  | **Y_20_** |  |  |  |  |
| --- | --- | --- | --- | --- | --- | --- | --- |
| **Variables** | **Total numbers** (n=1865) | **Y_0_**  **(%)** | **Lost to follow-up**  (n=617) | **Y_20_**  **(%)** | **Followed-up**  (n=1248) | **Y_20_**  **(%)** | **p-value** |
| **Sex** |  |  |  |  |  |  | ns |
| Women | 527 | 28.3 | 184 | 29.8 | 344 | 27.5 |  |
| Men | 1337 | 71.7 | 433 | 70.2 | 904 | 72.5 |  |
| **Age** |  |  |  |  |  |  |  |
| Age range | 18-93 |  | 18-93 |  | 18-78 |  | ns |
| Median, in total | 44 |  | 43 |  | 44 |  |  |
| Median age, women | 44 |  | 44 |  | 44 |  |  |
| Median age, men | 43 |  | 43 |  | 44 |  |  |
| **Living with another adult** (yes) | 443 | 23.8 | 146 | 23.7 | 297 | 23.9 | ns |
| **Work or studying in the past 30 days** (yes) | 363 | 19.5 | 117 | 19.0 | 246 | 19.8 | ns |
| **Substance dependence** (ICD 3+; based on CIDI) |  |  |  |  |  |  | ns |
| Alcohol, only | 1027 | 55.6 | 349 | 57.5 | 678 | 54.6 |  |
| Narcotic drug, only | 701 | 37.9 | 225 | 37.1 | 476 | 38.4 |  |
| Both alcohol and another drug | 93 | 5.0 | 26 | 4.3 | 67 | 5.4 |  |
| Neither alcohol nor another drug | 27 | 1.5 | 7 | 1.2 | 20 | 1.6 |  |
| **Recruited from/Index treatment** |  |  |  |  |  |  |  |
| Health care sector: |  |  |  |  |  |  | ns |
| Inpatient detoxification | 579 | 31.2 | 158 | 25.6 | 421 | 33.7 |  |
| MMT | 32 | 1.7 | 10 | 1.6 | 22 | 1.8 |  |
| Specialized unit (infections, dependence on pharmaceuticals) | 125 | 6.7 | 39 | 6.3 | 86 | 6.9 |  |
| Outpatient | 204 | 10.9 | 73 | 11.8 | 131 | 10.5 |  |
| Social services sector: |  |  |  |  |  |  | .031 |
| Outpatient (incl. assessments) | 632 | 33.9 | 212 | 34.4 | 420 | 33.7 |  |
| Residential care | 77 | 4.1 | 37 | 6.0 | 40 | 3.2 |  |
| Housing assistance | 193 | 10.4 | 80 | 13.0 | 113 | 9.1 |  |
| Compulsory care | 20 | 1.1 | 7 | 1.1 | 13 | 1.0 |  |
| **Treatment** for alc a/o drug problems (last year) | 1443 | 78.6 | 463 | 77.6 | 980 | 79.0 | ns |
| **Treatment** alc a/o drug problems (previous in life) | 1706 | 92.9 | 550 | 92.1 | 1156 | 93.2 | ns |
|  | **Y_0_** |  | **Y_20_** |  |  |  |  |
| *Cont.* | **Total numbers** (n=1865) | **Y_0_**  **(%)** | **Lost to follow-up**  (n=617) | **Y_20_**  **(%)** | **Followed-up**  (n=1248) | **Y_20_**  **(%)** | **p-value** |
| **Previous help** for alc a/o drug problems from social services | 253 | 13.6 | 99 | 16.0 | 178 | 14.3 | ns |
| **Compulsory care** (yes, previously) | 216 | 11.8 | 63 | 10.6 | 153 | 12.4 | ns |
| **Methadone** maintenance treatment (yes, previously) | 27 | 1.5 | 24 | 4.0 | 44 | 3.6 | ns |
| **Medication for psychiatric problems** (yes, previously) | 844 | 45.3 | 283 | 47.6 | 561 | 45.5 | ns |

^a^ Variables based on structured self-reported interview data

**Baseline interview measures**

Respondents were categorized by *primary substance* at baseline – i.e., dependence on alcohol, dependence on another illicit/prescription drug, both alcohol and another drug, or not meeting diagnostic criteria for dependence. The background variables *sex*, *age*, *living with another adult*, *working or studying*, whether *recruited from* the health care system or social services, *treatment* in the last year (prior to baseline) and in the past, *previous help* from social services, as well as previous experience of *compulsory care*, *methadone maintenance (OAT)*, and *medication for psychiatric problems* were derived from the baseline interview data.

| **VARIABLE** | **OPERATONALIZATOIN** |
| --- | --- |
| *Living with another adult* | What is your marital status? (from the Addiction Severity Index; ASI)   1. Married 2. Live together with partner 3. Alone, have been married or have lived together with a partner 4. Alone, have never been married or lived with a partner |
| *Work or studying in the past 30 days* | Option 1 and 2 in SF8. Which is the major source of your support? (ASI)   1. Employment (include “black work”) 2. Study allowance 3. Unemployment compensation 4. Public assistance or welfare 5. Sickness-benefit, pension 6. Partner, family or friends 7. Illegal activity 8. Prostitution 9. Other |
| *Substance dependence* | Questions from CIDI (36) were used to calculate the number of ICD-10 (37) alcohol dependence criteria for alcohol. Those scoring 3+ out of the six criteria were categorized as dependent on alcohol at baseline.  Individuals who also had been using another illicit drug in the past twelve months were asked “which kind of drug or medication have you used most often in the past 12 months”, if the person primarily came to treatment for alcohol problems. Research participants that reported that they had entered treatment for their illicit drug use were asked “Which of these kinds of drugs or medications is the main reason you are coming to treatment?”. The categories for main drug of choice were: opioids, sedatives, cocaine, amphetamines, cannabis, hallucinogens, solvents. Next, the participant was asked the CIDI/ICD-10 questions for his/her main illicit drug of choice.  Participants were thereby categorized as either dependent on alcohol only, dependent on an illicit or prescription drug only, dependent on both alcohol and another drug, or not scoring 3+ on either alcohol or drug dependence. It is important to note that this is a clinical sample, which means that everyone had some kind of substance-related problem. However, not everyone met the diagnostic criteria for alcohol or drug dependence at baseline. |
| *Recruited from/Index treatment* | The research team tracked recruitment and index treatment. In the health care-based SUT system, patients were recruited from several inpatient detoxification wards, the methadone maintenance program (the only OAT program available at that time), two specialized units for the treatment of dependence on pharmaceuticals (benzodiazepines, painkillers like codeine, etc.) and infectious diseases among substance users, and finally from various outpatient services. Clients were recruited from the social services’ SUT in a sample of municipalities and city districts selected to be representative of the region. Outpatient clients were either undergoing an assessment to decide on a treatment option, had already been granted an outpatient program, or had ongoing contact with the social worker regarding AOD. Residential care refers to long-term care in specialized units run by the municipality or another contracted service provider. Housing assistance usually referred to facilities for AOD users while participating in treatment. Compulsory care of up to six months is assessed by the social services, decided by the administrative court and provided in state institutions. |
| *Treatment for AOD problems* | Participants previous experiences of treatment referred to ‘all treatment, care or other support you have received in your life and in the past 12 month that had to do with your alcohol or drug use’. Twenty categories were checked, the first 10 referring to health care-based services, 6-10 to social services, 7 to compulsory care, 8-9 to the criminal justice system, and finally to self-help groups, and other. The response categories were: never, previously, in the last 12 months. Those who had received such care in the past year were also asked for the number of days in such care.   1. Acute inpatient treatment (TNE in BCN [Dependence centre north], ward 9 in BCS [Dependence centre south]) 2. Detoxification or other inpatient treatment in BCN or BCS 3. Outpatient unit in BCN or BCS 4. Mobile team (health system) 5. Methadone-program 6. Inpatient treatment, psychiatric sector 7. Outpatient unit, psychiatric sector 8. Other inpatient treatment, hospital (health system) 9. Other general outpatient treatment in the health system 10. Private health care/ psychologist or therapist 11. Intensive outpatient treatment arranged by the social services (>6 hours/week) 12. Other outpatient treatment arranged by the social services (<6 hours/week) 13. Therapeutic community/recovery home (excl. compulsory treatment) 14. Housing support (excl. shelters) 15. Work training 16. Compulsory treatment (LVM – for adults) 17. Outpatient treatment ordered by probation, e.g., drinking driving program 18. Treatment in prison 19. AA, NA, Links and other self-help groups. 20. Other, e.g., family care, outreach groups, shelters |
| *Previous help from social services* | SUT from social services summarizes whether the participant had previously received treatment through social services, categories 11-15 above. |
| *Compulsory care* | Category 16 above. |
| *Methadone maintenance* | Category 5 above. |
| *Medication for psychiatric problems* | This measure used an ASI question for psychiatric status: Have you been prescribed medications for any psychological or emotional problems? Either in the past 30 days, previously, or never. The question referred to periods when the participant was not under the direct influence of alcohol or drugs. |

1. Sohlberg, Storbjörk, Wennberg. Measuring social integration, treatment, and mortality after substance use treatment: methodological elaborations in a 20-year follow-up study. References are found in the article. [↑](#footnote-ref-1)
2. We adopt slightly different labels than Alm (16) and the NBHW (49) since many in our sample have exited the labor market and have a pension at Y_20_. [↑](#footnote-ref-2)
